# Evaluation of Hospital Antimicrobial Stewardship Programs: Implementation, Process, Impact, and Outcomes, Review of Systematic Reviews

**DOI:** 10.3390/antibiotics13030253

**Published:** 2024-03-12

**Authors:** Hamad Abdel Hadi, Faiha Eltayeb, Sara Al Balushi, Joanne Daghfal, Faraz Ahmed, Ceu Mateus

**Affiliations:** 1 Communicable Diseases Centre, Hamad Medical Corporation, Doha P.O. Box 3050, Qatar; salbalushi3@hamad.qa (S.A.B.); jnader@hamad.qa (J.D.); 2 Division of Health Research, Faculty of Health and Medicine, Lancaster University, Lancaster LA1 4YW, UK; faraz.ahmed@lancaster.ac.uk (F.A.); c.mateus@lancaster.ac.uk (C.M.); 3 Division of Microbiology, Department of Laboratory Medicine and Pathology, Hamad Medical Corporation, Doha P.O. Box 3050, Qatar; feltayeb1@hamad.qa

**Keywords:** Antimicrobial Stewardship Programs (ASP/AMSP), antimicrobial consumption, antimicrobial resistance

## Abstract

Antimicrobial Stewardship Programs (ASP) were introduced in healthcare as a public health priority to promote appropriate prescribing of antimicrobials, to reduce adverse events related to antimicrobials, as well as to control the escalating challenges of antimicrobial resistance. To deliver aimed outcome objectives, ASPs involve multiple connected implementation process measures. A systematic review was conducted to evaluate both concepts of ASPs. Guided by PRISMA frames, published systematic reviews (SR) focusing on ASPs restricted to secondary and tertiary healthcare were evaluated over the past 10 years involving all age groups. Out of 265 identified SR studies, 63 met the inclusion criteria. The majority were conducted in Europe and North America, with limited studies from other regions. In the reviewed studies, all age groups were examined, although they were conducted mainly on adults when compared to children and infants. Both process and outcomes measures of ASPs were examined equally and simultaneously through 25 different concepts, dominated by efficacy, antimicrobial resistance, and economic impact, while information technology as well as role of pharmacy and behavioral factors were equally examined. The main broad conclusions from the review were that, across the globe, ASPs demonstrated effectiveness, proved efficacy, and confirmed efficiency, while focused evaluation advocated that developed countries should target medium- and small-sized hospitals while developing countries should continue rolling ASPs across healthcare facilities. Additionally, the future of ASPs should focus on embracing evolving information technology to bridge the gaps in knowledge, skills, and attitude, as well as to enhance appropriate decision making.

## 1. Introduction

The primary goals of public health (PH) are to improve population wellbeing and outcomes through promotion of health, prevention of diseases, and facilitation of fair access to healthcare [1]. Fundamental to the concept of PH is the provision of safe and quality care which is equitable and cost-effective [2]. To achieve such targets, efforts should be directed towards reliable mechanisms for the evaluation of healthcare program interventions [3]. Historically, aims were directed towards efficacy and outcomes; however, it has been argued that achieving the intended objectives does not always equate to delivering quality care, since, according to Linford et al., outcomes measures are “blunt instruments for judging performance” [4].To bridge that gap, quality experts recommend the provision of appropriate evaluation methods to facilitate the delivery of aimed objectives [4]. Therefore, programs evaluation is of utmost importance primarily to appraise effectiveness and desired outcomes, as well as to address challenges [5]. According to the Centers for Disease Control and Prevention (CDC) of the USA, the process is defined as: “a systematic method for collecting, analyzing, and using data to examine the effectiveness and efficiency of programs and, importantly, to contribute to continuous program improvement” [6]. 

Alarmed by concerns regarding inappropriate over-prescribing of antimicrobials in healthcare, which is directly linked to adverse events in patients as well as being indirectly associated with escalating antimicrobial resistance (AMR), at the turn of the 21th century, Western healthcare authorities across the Atlantic introduced the concept of Antimicrobial Stewardship Programs (ASPs) following a justifiable appeal from experts in the field [7,8]. According to the CDC, at inpatient hospital settings, more than 50% of prescribed antimicrobials are not consistent with the recommended practice, while the majority of common infections are over-treated [6]. Comparably, the National Institute for Healthcare and Excellence (NICE) in the UK defines the process as: “the organizational or healthcare-system-wide approach to promote and monitor the judicious use of antimicrobials to preserve their future effectiveness” [9]. The program entails the collaboration of a wide range of healthcare professionals led by infection and antimicrobial specialists with the prime aim to oversee appropriate and optimal prescribing of antimicrobials through the provision of guidance and awareness, in addition to monitoring of outcomes [10].

Almost twenty-five years following their implementation, ASPs became widely accepted and subsequently embraced at national and international levels. They were eventually promoted by the WHO to the point of being hailed as one of the major 21st century public health interventions [11,12,13]. Nevertheless, because of differences in healthcare settings and population diversity, there have been uncertainties regarding conclusive and generalizable evidence to answer raised questions about topics such as efficacy and outcomes [13,14]. For example, there are implementation challenges because of local epidemiology or healthcare settings [15,16], conflicting differences for optimal program elements [14,17], the key role of antimicrobial pharmacists [18], the paucity of reporting of microbiological outcomes [19], and challenges in surveillance processes [14]. Similarly, program outcomes such as targeted mortality has been conflicting with reports citing lower rates, while others show no differences [20,21]. Conversely, despite multiple studies concluding that ASPs can reduce AMR [22,23,24], others were not conclusive [20,25]. Equally, the economic impact of the program is confounded by difficulties in adopting consensus models [11,26]. Lastly and importantly, although the program has major components, some studies have looked at the additional value of information technology (IT), reinforcing the benefits of auxiliary concepts, such as the use of smart applications, that merit further evaluation [27,28]. 

For all these reasons, it is prudent to have an umbrella overview to evaluate ASPs’ process measures, impacts, and outcomes, as well as assess any specific service improvement concepts. Distinctively, although other systematic reviews, meta-analyses, and overviews have examined different concepts of ASPs repeatedly, they only focused on specific aspects of the program rather than overall comprehensive evaluation [25,29,30]. 

## 2. Methods and Search Strategies

A systematic literature review was conducted, guided by the framework of Preferred Reporting Items for Systematic reviews and Meta-Analyses (PRISMA) [31]. We included only peer-reviewed primary systematic reviews (SRs) in ASPs that reported any implementation process, impact, or outcome measures specifically at inpatient hospital settings encompassing secondary and tertiary healthcare settings, with no age restrictions. Five major databases were searched: OVID-Medline, PubMed, Embase, Cochrane, and Google Scholar. The search was conducted and updated up to 28 August 2022 and was restricted to the last 10 years, focusing on humans with no language restrictions. Studies that were conducted at primary, ambulatory, or long-term facilities were excluded, together with studies that focused on singular countries or pathogens. Similarly, studies with projected titles and methods focused mainly on reviews or scoping reviews with non-congruent systematic methodology were excluded. Since the COVID-19 pandemic is still unfolding, related data on the subjects were excluded. Guided by inclusion and exclusion criteria, two separate primary investigators screened titles, abstracts, and methods and agreed on a final output when selected studies were read in depth, including ranking for critical appraisal. 

## 3. Search Outcomes

**Figure 1:**
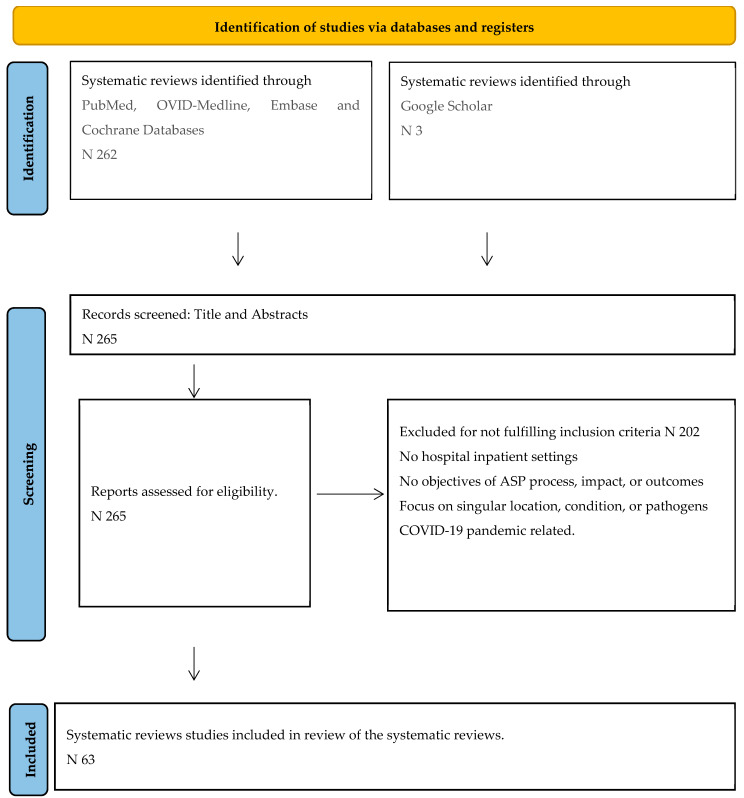
PRISMA flow diagram showing search steps of identification, screening, and final inclusion.

The search outcome is depicted as recommended in PRISMA Figure 1, and details of search protocols are provided in Supplementary Material. Out of 265 identified SRs, 202 studies were excluded, leaving the remaining 63 studies for final analysis.

## 4. Synthesis of Evidence

Since studies were heterogenous in their concepts and methods, examining wide aspects of the program, thematic categorization of studies was performed to produce subclassifications according to the published reporting period, the origin of affiliated institutions, the global or regional focus of the studies, the studied population, and, more importantly, objective concepts. The six recognized WHO geographical criteria were used to group regional countries accordingly [32]. Since the concepts of effectiveness (adopting correct program approaches), efficacy (obtaining aimed outcomes), and efficiency (obtaining outcomes through cost effectiveness) are at the heart of healthcare, they were adopted in categorizing the concepts of ASPs [33]. Likewise, elements of process measures (implementation facets such as the role of antimicrobials guidelines) and elements of outcome measures such as antimicrobial consumption metrics were similarly examined. Additionally, historic concepts of ASPs such as closely linked AMR and quality indicators such as safety outcomes, represented by reducing adverse events, were similarly adopted, as well as other program aspects such as behavioral interventions, microbiological outcomes, and the roles of pharmacy and IT (Table 1 and Table 2). From each examined theme, specific conclusions were extracted together with relevant recommendations.

## 5. Critical Appraisal for Quality of Evidence

Critical appraisal of the examined SRs was conducted by two investigators using the updated online version of A Measurement Tool to Assess Systematic Reviews (AMSTAR-2) [34]. Studies were ranked from high to critically low depending on the overall non-numerical assessment. It must be emphasized that, since the concepts of ASPs are complex, with clear heterogeneity that is frequently not tested through desired evidence-based practice, including randomized trials or meta-analysis with no rigorous safeguarding for biases, it was not surprising that the stringent AMSTAR-2 critical appraisal criteria rated most SRs as low-quality, with few exceptions Table 1 [20,35].

**Table 1:** Systematic review studies classified according to first author, year of publication, publishing institution according to the WHO regional classification, number of evaluated studies, primary studied concepts, examined process and outcome measures as well as critical appraisal of quality of evidence.

Regions.	Systematic Reviews/Year	Approach	Primary Concept	Studies	Process Measures	Outcome Measures	* AMSTAR-2 Quality	Ref.
Europe (EUR)	Baur 2017	Global	Adverse Events	32	No	Yes	CL	[36]
	Chatzopoulou 2020	Global	Antimicrobial Resistance	15	Yes	Yes	CL	[37]
	Chatzopoulou 2022	Global	Antimicrobial Resistance	29	Yes	Yes	CL	[38]
	Corafa 2022	Global	Critical Care	13	Yes	Yes	CL	[39]
	Davey 2013	Global	Effectiveness	89	Yes	Yes	L	[20]
	Davey 2015	Global	Behavior	116	Yes	No	CL	[40]
	Davey 2017	Global	Safety and efficacy	221	Yes	Yes	H	[20]
	Donà 2020	Global	Efficacy	113	No	Yes	CL	[41]
	Dik 2015	Global	Economic impact	95	Yes	No	CL	[26]
	Helou 2020	Global	Information Technology	13	No	Yes	CL	[28]
	Huebner 2019	Global	Economic Impact	16	Yes	No	CL	[42]
	Kallen 2017	Global	Quality Indicators	14	No	Yes	CL	[43]
	Lau 2022	Global	Microbiological outcomes	117	Yes	Yes	CL	[19]
	Mas-Morey 2018	Global	Role of Pharmacy	28	Yes	No	CL	[44]
	Micallef 2017	Global	Information Technology	14	Yes	Yes	CL	[45]
	Monmaturapoj 2021	Global	Role of Pharmacy	52	Yes	Yes	CL	[46]
	Monnier 2018	Global	Quality Indicators	70	No	Yes	CL	[47]
	Nathwani 2019	Global	Economic outcomes	164	Yes	Yes	CL	[48]
	O’Riordan 2021	Regional	Quality Indicators	16	Yes	Yes	CL	[49]
	Porter 2021	Global	Behavioral Factors	14	Yes	No	CL	[50]
	Pouly 2022	Global	Behavioral Factors	124	Yes	Yes	CL	[51]
	Rajar 2021	Global	Safety	12	Yes	No	H	[35]
	Rawson 2017	Global	Information Technology	58	Yes	No	CL	[52]
	Rzewuska 2020	Global	Implementation	145	No	Yes	CL	[53]
	Schuts 2016	Global	Efficacy	15	Yes	Yes	CL	[54]
	Schuts 2021	Global	Antimicrobial Resistance	145	Yes	No	L	[25]
	Schweitzer 2019	Global	Quality of studies	825	Yes	No	CL	[55]
	Stanic 2018	Global	Metrics	168	Yes	Yes	CL	[56]
	Tacconelli 2016	Global	Surveillance	78	Yes	Yes	CL	[14]
	Van Dijck 2018	Global	MLIC *	27	Yes	No	CL	[57]
	Warreman 2019	Global	Behavioral Factors	9	Yes	No	CL	[58]
Americas (AMR)	Araujo Silva 2018	Global	Effectiveness	9	Yes	Yes	L	[59]
	Bertollo 2018	Global	Antimicrobial Resistance	26	No	Yes	CL	[60]
	Daniels 2021	Global	Discharge medications.	6	Yes	No	CL	[61]
	Feazel 2014	Global	Adverse Events	78	No	Yes	L	[62]
	Karanika 2016	Global	Economic Impact	26	No	Yes	CL	[29]
	Kooda 2022	Global	Role of Pharmacy	24	Yes	Yes	L	[18]
	Lindsay 2019	Global	Critical care	11	No	Yes	CL	[21]
	Losier 2017	Global	Emergency Department	43	Yes	No	CL	[17]
	Murray 2021	Global	Antimicrobial Resistance	29	No	Yes	CL	[63]
	Rennert-May 2017	Global	Guidelines	5	Yes	No	CL	[64]
	Rittmann 2019	Global	Information Technology	45	Yes	Yes	CL	[65]
	Smith 2015	Global	Efficacy	9	No	Yes	CL	[66]
	Wade 2021	Regional	HCAIs **	21	Yes	Yes	CL	[67]
	Wagner 2014	Regional	Efficacy	37	Yes	Yes	CL	[68]
West Pacific Region (WPR)	Abo 2020	Global	Efficacy	34	Yes	Yes	L	[69]
	Baysari 2016	Global	Information Technology	45	Yes	No	CL	[27]
	Honda 2017	Regional	Safety and efficacy	46	No	Yes	CL	[16]
	Lee 2018	Regional	Safety and efficacy	77	No	Yes	CL	[70]
	Lim 2020	Global	National Interventions	34	Yes	No	CL	[71]
	Roman 2018	Global	Role of Pharmacy	15	Yes	No	CL	[72]
	Siachalinga 2022	Global	Efficacy	28	Yes	No	L	[73]
	Tabah 2016	Regional	Critical Care	14	Yes	Yes	CL	[74]
East Mediterranean (EMRO)	Ababneh 2021	Regional	Implementation	20	Yes	Yes	CL	[15]
	Atamna 2021	Global	Antimicrobial Resistance	63	Yes	Yes	CL	[75]
	Bitterman 2016	Global	Antimicrobial consumption	80	Yes	No	CL	[76]
	Garwan 2022	Global	Antimicrobial Switch	36	Yes	Yes	CL	[77]
	Hashad 2020	Regional	Effectiveness	17	Yes	No	CL	[78]
	Keikha 22	Global	Antimicrobial Resistance	17	No	Yes	L	[79]
	Nasr 2017	Regional	Behavioral factors	20	Yes	No	CL	[80]
Southeast Asia (SEAR)	Ibrahim 2017	Global	Economic Impact	5	No	Yes	CL	[81]
	Teerawattanapong 2017	Global	Antimicrobial Resistance	42	Yes	Yes	CL	[82]
Africa (AF)	Akpan 2020	Regional	Implementation	13	Yes	Yes	CL	[83]

* AMSTAR-2: Measurement Tool to Assess Systematic Reviews, evaluation tool to review the methodological quality of systematic reviews: H; high-quality review, M; moderate, L; low, CL; critically low. * MLIC: middle- and low-income countries. ** HCAIs: healthcare-associated infections.

## 6. Results

**Figure 2:**
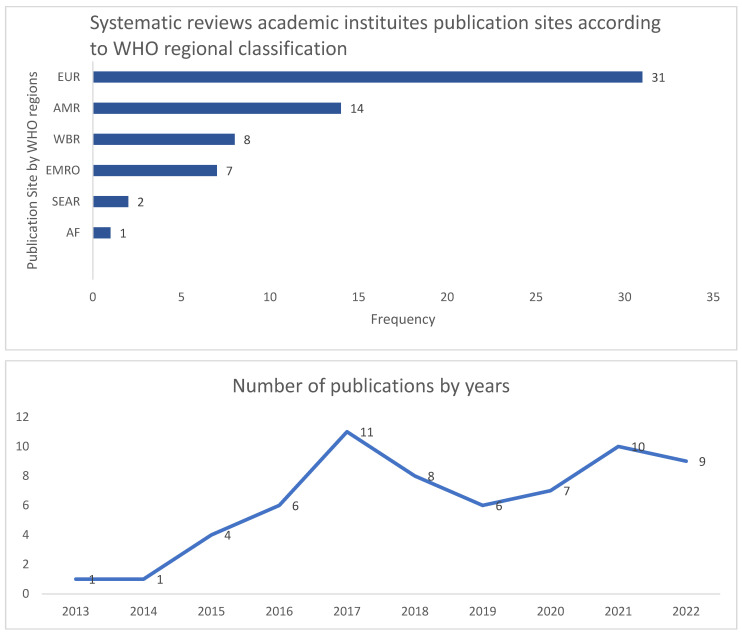
Systematic review publication sites according to the WHO regional classifications and cumulative records of publication year. EUR: European region, AMR: Region of Americas, WPR: Western Pacific Region, EMRO: Eastern Mediterranean Region, SEAR: Southeast Asian Region, and AF: African Region.

Out of 63 selected systematic reviews, the majority were conducted in the European region (EUR: 31) and regions of the Americas (AMR: 14) at a total of 71% (46/63), while the remaining regions constituted 29% (18/63), detailed as: Western Pacific region (WPR:8), Eastern Mediterranean Region (EMRO: 7), Southeast Asian Region (SEAR: 2), and African Region (AF:1), as outlined in Figure 2. Most of the SRs (84%) focused on global objectives, while only 16% reported regional data. Predominant SRs (51/63) included all age groups, while adults were specified in 6, children in 5, and infants in only 1 study. The ASPs were evaluated through 25 different concepts, dominated by efficacy and safety (9) and AMR as outcomes (8), while economic impact was evaluated in (5) studies, the role of information technology in (5), as well as the role of pharmacy (4) and behavioral factors (4), as outlined in Table 2. Out of the 63 SRs, 22 (35%) solely examined the implementation process, while 17 (27%) focused on outcome measures, and the two concepts were examined in 38%.

**Table 2:** Primary examined concepts for the systematic reviews.

Primary Focus	Frequency	Percentage
Antimicrobial Resistance	8	12.7
Efficacy	6	9.52
Behaviour	5	7.94
Information technology	5	7.94
Economic impact	4	6.35
Quality	4	6.35
Role of Pharmacy	4	6.35
Critical Care	3	4.76
Effectiveness	3	4.76
Efficacy and Safety	3	4.76
Implementation	3	4.76
Adverse Events	2	3.17
Antimicrobial Switch	1	1.59
Antimicrobial consumption	1	1.59
Clinical and economic outcomes	1	1.59
Discharge medications	1	1.59
Emergency department	1	1.59
Guidelines	1	1.59
Healthcare Associated infections	1	1.59
Metrics	1	1.59
Microbiological outcomes	1	1.59
Middle- and low-income countries	1	1.59
National interventions	1	1.59
Safety	1	1.59
Surveillance	1	1.59
Total	63	100%

## 7. Overview of Evaluation Process

Examining the spectrum of global studies, most SRs focused on Western populations, with a paucity of data from developing countries (Figure 2). In their large and highly rated SR focusing on efficacy and safety, the study of Davey et al. encompassed 221 articles, including 58 randomized controlled trials and 163 studies of other kinds: Most of the studies were from North America (96) and Europe (87), while the remaining were from Asia (19), South America (8), Australia (8), and East Asia (3) [20] (Table 1 and Figure 2). 

## 8. Studied Population

The ASP systematic reviews identified the studied populations as adults, children, and infants mainly from high income Western countries [20,41,66]. Most of the data from the SRs were extracted from adult populations, while data from the pediatric cohorts were scarce, especially regarding the implementation measures (Table 2) [19,66]. Despite the limited available data from the pediatric population, the ASPs managed to implement objectives without compromising safety, even at pediatric intensive care units (PICUs), with limited data regarding healthcare-associated infections and antimicrobial resistance [66]. 

## 9. Healthcare Settings

Although the majority of secondary and tertiary care facilities in developed countries are small- and medium-sized hospitals (200–500 beds), the majority of data were extracted from studies conducted at large-sized hospitals (>500 beds), where antimicrobial consumption might be similar, but the implementation barriers are more pronounced [11,84]. Conversely, despite the highlighted benefits of the program in different regions across the globe, there are equally outlined implementation and outcome challenges in developing regions, leading to strong demands to strengthen fundamental aspects of the program [12,15,16,83]. Therefore, to improve global delivery of ASP objectives, healthcare services in developed countries should focus their attention towards small- and medium-sized hospitals, while developing countries should cement ASPs’ core elements. 

## 10. Process Implementation Interventions

The main examined interventions during program implementation include empirical therapy according to guidelines, timely de-escalation therapy, switching from intravenous to oral, therapeutic drug monitoring, and preauthorization through restricted antimicrobial lists. From multiple SRs, there is evidence of successful implementation of these interventions aligned with outcomes [16,20,25,29]. When comparing enablement objectives with or without ASP interventions, there were no observed mortality differences [20]. Efficacy without compromising safety has been similarly observed in vulnerable populations at critical care units, in both adult and pediatric settings, through effective program interventions such as prescribing audits, feedback, education, and persuasion [20,21,39].

## 11. Epidemiology and Surveillance

Although the importance of surveillance and epidemiological reports in guiding ASPs has been clearly overstated, from multiple SRs, challenges are evident in both developed and developing countries with variable representation [14,15,16]. Moreover, although ASPs have been implemented with anticipations to curb AMR as an outcome, SR studies have described challenges in accurate reporting in healthcare affecting accuracy [25,60]. Additionally, there is a paucity of studies that report accurate microbiological results linked to ASPs, which is the hallmark for monitoring AMR [19]. To bridge such gaps, it is advocated to conduct proper AMR surveillance methods in all healthcare facilities that should guide the ASP implementation process, and not vice versa [14,85]. 

## 12. Efficacy and Safety

Since the program’s concepts are based upon restricting antimicrobials as well as reducing the duration of therapy, there have been some concerns that it might impact patients’ safety. From multiple SRs, ASPs have proven to be efficacious in reducing antimicrobial consumption and inappropriate prescribing without compromising patients’ safety, for both adult and pediatric populations [20,41,48,59]. The feared prime concerns were understandably directed towards critical care, where the highest levels of hospital antimicrobial consumption are usually reported, combined with the critical nature of the patients’ cohort. Nevertheless, SRs in such important aspects affirmed safety and efficacy, although the evidence is more pronounced for adult when compared to pediatric populations because of limited data [21,39,59,74]. However, the picture is not fully clear, since there is a counterargument that restricting antimicrobials at critical care settings might be protective through limiting unfavorable adverse events or might be confounded by the plausible practice of reducing interventions in less critical patients [74]. 

## 13. Influencing Prescribing Behavior

The foremost definition of ASPs accentuates “appropriate and judicious prescribing of antimicrobials”, which entails focusing efforts to influence the behaviors and attitudes concerning antimicrobial prescribing directed towards healthcare professionals [20]. Determinant factors imported from behavioral studies, such as psychosocial theory of planned behavior, affect how developed cognition influences decision making and behavior [58,86]. In ASPs, the main positive attitude determinants that influence prescribing behavior have been identified as education and training, as well as audit and feedback aimed to alter conceived prescribing cultures [20]. Despite the conceivable association between the influence of cognitive behavioral factors and previous experience on prescribing cultures, some SRs have pointed towards gaps in targeting outlined attitudes, particularly the lack of qualitative studies, to address raised barriers. For such reasons, exploring prescribing attitudes and practices, particularly behavior change techniques, is encouraged in both developed and developing countries [20,50,58]. 

## 14. Quantifying Metrics

Appropriate quantifying metrics (QM) must be adopted to closely monitor consumption as well as to estimate associated costs. As the concept of ASPs was introduced without defining relevant QMs, subsequent mounting challenges were to evaluate existing ones as well as trying to develop new methods [87]. Amongst the existing QMs were the Defined Daily Dose (DDD), which was developed in the late 1970s and standardized through patients’ total hospital days, depicted per patient days (DDD-100 or 1000 patients days) [76]. In their SR regarding QMs, Stanic Benic et al. identified 12 common measurements that include the commonly used DDDs, Days of Therapy (DOT), as well as the total antimicrobial cost. The recommended practice is a combined metric approach using at least two of the prime measures [56]. Although there have been many cited limitations for using QMs such as DDDs because of variations in relation to geographical locations or hospital settings, the role of weight adjustments, and adjusted doses in renal impairment as well as combined therapy, it has been widely adopted across the globe in the adult population, as well as being recommended by the WHO [88]. Because of multiple factors dominated by patients standard weight, in the pediatric population, metric challenges have been identified with no optimal recommended measures, although DOT is generally preferred when compared to DDDs [89]. 

## 15. Role of Infection Prevention and Control

Although not examined as a primary specific concept of the program in recent SRs, mutiple studies have covered the alliance of Infection Prevention and Control (IPC) programs with the parallel and closely related ASPs, frequently featured as influencing both the process and outcome measures of each other [90]. Since the main objective of IPCPs is to limit the spread of infection across healthcare facilities, including communicable diseases as well as multidrug-resistant organims (MDROs), it is not surprising that failing in that domain will ultimately affect ASPs through subsequent secondary increases in antimicrobial prescribing and, hence, consumption. Likewise, the successful implementation of ASPs can directly facilitate the aims and objectives of IPCPs. These plausible observations of utmost synergy are supported by multiple infection societies and organizations that promote both concepts [91]. In their SR study, Buar et al. demonstrated that ASPs can reduce Clostridium difficile infections (CDIs) by almost 50%, thus positively impacting on the delivery of IPCPs [36]. Furthermore, one of the main outcome measures of ASPs is reducing rates of AMR consequences such as the acquisition of secondary MDROs infections [79,92]. Hence, poor implementation of IPC measures can directly lead to the ultimate failure of ASP outcomes. Correspondingly, in their recommendations to optimize the reporting of epidemiological studies for antimicrobial resistance to improve the implementation of ASPs, Tacconelli et al. advocate for the optimal implementation of IPC measures to limit the spread of MDROs across healthcare facilities [14]. Similarly, in their high-ranking SR, Davey et al. identified IPCPs as one of the fundamental interventions for the successful implementation of ASPs [20]. Furthermore, the cornerstone role of IPC in ASP has also been similarly oulined for the pediatric population [59]. Although the interface of ASPs and IPCPs is closely related with difficult-to-measure outcomes, we advocate the expansion of research on this vital aspect to guide effective interventions. 

## 16. Role of Pharmacy

Few SRs have examined the roles of antimicrobial pharmacists during the implementation of ASPs, demonstrating effective interventions in the form of education-based interventions, compliance with guidelines, and reductions in the duration of antimicrobial therapy, calling for further empowerment [46]. Similarly, the role of pharmacists has been emphasized in the emergency department as well as in small- and medium-sized hospitals, where pharmacists play a major role in ensuring appropriate prescribing and guarding against misuse [18,44]. Uniquely, Daniels and Weber looked at the role of hospital-based pharmacists in verifying hospital discharge antimicrobials, highlighting positive outcomes in validating antibiotic choice, duration, frequency, and directed therapy in line with ASP objectives [61]. While reviewing the role of pharmacy in ASPs, it must be emphasized that the recommendations from the WHO since 2017 have been to safeguard against the development of AMR and help to encourage the successful implementation of ASPs by adopting specific prioritization of antimicrobials. The concept advocates for classifying antimicrobials into three categories: essentials, with lower potential barriers for AMRs (Access); critically important, with potential impacts on resistance (Watch); and last-resort antimicrobials to combat the challenges of MDROs (Reserve). These are combined in the acronym AWaRe [93]. Although the benefits of the recommended classification have been advocated globally, the need for raising awareness about the concept has been emphasized more for developing countries, particularly when facing antimicrobial cost constraints [94]. 

## 17. Evaluation of Outcome Measures

### 17.1. Interface of ASP and Its Impact on Antimicrobial Resistance

The global and secondary care burden of antimicrobial resistance on morbidity, mortality, and cost of management has been previously outlined in major studies [95,96]. Despite the plausible link between consumption of antimicrobials and the development of AMR, the causal relationship has proved to be difficult to establish. In their SR encompassing 32 studies and almost 9 million reviewed reported cases, Baur et al. reported that ASPs reduce hospital infections and colonization with MDROs and CDI by a factor of almost 50%. A similar outcome of reduction in infections caused by MDROs was shared by Karanika et al. who added that reduced antimicrobial consumption was not associated with observed adverse outcomes [29,36]. This view is contradicted by Tacconelli et al., who highlighted flaws with epidemiological reports, while Chatzopoulou et al. pointed that the plausible projected hypothesis is supported by poor evidence, in addition to Bertollo et al. citing the paucity of randomized controlled trials and reliance on observational and quasi-experimental studies [14,37,60]. The lack of conclusive evidence to support direct correlation has been affirmed by a wider meta-analysis conducted by Schuts et al. [25]. 

When trying to delve into potential explanations for the lack of supporting evidence despite the coherent assumption, there are major confounding factors which are impossible to control. For example, potential factors that might directly affect the propagation of AMR include the local environment and various healthcare settings and the demographics of the affected populations, including susceptibilities to infections, either from acquired or genetic factors or colonization with resistant strains; previous exposure to antimicrobials; dominance of certain resistant clones; and access to critical care, including the use of invasive devices as well as local practices of infection control and prevention [14,97,98]. Hence, to bridge the obvious gap between the implementation of ASPs and the reduction in AMRs in healthcare settings, it is conceivable to advocate for reliable and functional epidemiological reporting and surveillance systems, which must be continuously improved and developed [14].

### 17.2. Economic Impact and Cost Effectiveness 

Although it was not declared amongst the main goals of ASPs at its inception, it was soon realized that there are observed secondary benefits based upon economic savings [8]. From reciprocated SRs, the economic benefit of the ASPs is evident mainly through three main aspects: direct saving from restricting more expensive broad-spectrum antimicrobials, reduction in the duration of therapy, and reducing patients’ length of hospital stay [11,26,29,42]. Nevertheless, there were conclusive remarks that there are non-uniform agreements for calculating economic parameters, the quality of extracted data, and the reported heterogeneity of studies in the pediatric population [55,66,81]. 

### 17.3. Quality Assessment and Development of ASPs 

In healthcare, quality has been defined as “the systemic process to improve healthcare delivery”, which is integral to the provision of continuously developed ASPs [99]. In their SR, Schweitzer et al. concluded that the overall studies in ASPs are of low quality and have not improved over time, and additionally, they expressed concerns regarding the lack of microbiological and clinical data as an outcome, calling to improve studies’ methodologies [55]. To converge towards a consensus on quality indicators for ASPs, about 50 QIs have been outlined to serve towards future standardized measures [47]. Challenges regarding quality in reporting and surveillance have been outlined, calling for standardized methods in both developed and developing countries [14,16].

## 18. Role of Modern Information Technology

Since the ASPs heavily rely on updated knowledge, accurate prescribing, and applied and retrieved data, it is plausible to explore the role of information technology (IT) towards improvements in the quality of the program [100]. The concept has been explored by Baysari et al., who identified computerized decision support system (CDSSs), computerized antimicrobial approval systems, and surveillance methods, concluding that there are positive outcomes confounded by the absence of comparative studies [27]. Although the modern role of CDSSs has become more evident in ASPs, Rawson et al. expressed concerns that it was not designed properly to guide antimicrobial prescribers, calling for better models [52]. Furthermore, Micallef et al. advocated for the secondary use of information technology and hospital electronic systems to support the delivery of effective ASPs [45]. Lastly, although not supported by much updated evidence, specific ASP smartphone applications to improve knowledge, skills, and attitudes towards antimicrobial prescribing have been demonstrated as a promising platform that is worth exploring [28]. 

## 19. Limitations

One of the fundamental limitations of the evaluation of the SRs regarding ASPs is that the prime concept is relatively new in different parts of the world, with less than 25 years of experience with continuously evolving standards. From the review, most of the data have been obtained from established Western healthcare facilities, with limited comparators from global developing countries. Additionally, from multiple SRs, extracted evidence has mainly been obtained from observational studies rather than the gold standards of randomized controlled or interventional trials with no comparators, dwarfing generated evidence and conclusions. Even upon review of studies examining shared concepts such as AMR or economic burden, there is clearly highlighted heterogeneity in the studies, objectives, and methodology, and no consensus in agreed-upon measurements, making combining evidence synthesis a daunting task. These limitations are undoubtedly reflected in the quality of evidence extracted from SRs examined through the AMSTAR critical appraisal tool, which demonstrated uniformly low-quality standards. Furthermore, in reviewing the concept of ASPs, there are multiple co-dependent confounding factors that can affect implantations and outcome processes. For example, facilities related to infection prevention and control program practices can directly impact both implantation processes, such as increased antimicrobial consumption, and outcomes by spreading AMR. Additionally, local population characteristics, healthcare settings, resources, and facilities, as well as professional practice culture, have direct effects on ASPs. 

## 20. Summary and Conclusions

This review of the SRs evaluating the implementation processes, impacts, and outcomes of ASPs has delineated that the concepts are strongly cemented in Western healthcare facilities but are evolving in developing countries. The studied population mainly consists of adults from Western countries, with limited data from children and infants. The process of implementation of effectiveness consists of multiple connected concepts ranging from guidelines and restricted antimicrobials to the roles of IT and pharmacists to influence prescribers’ behaviors. For safety and efficacy, objective interventions were met in all evaluating studies including vulnerable populations such as children, as well as patients under critical care. As for efficiency, evaluation of the economic impact of ASPs as a secondary outcome demonstrated proven benefits, mainly through reducing the expenditure of antimicrobial therapy as well as reducing the length of patients’ hospital stays. Although one of the fundamental aims of the ASPs is to reduce the mounting scale of AMR, the evaluated evidence does not conclusively support the objective opening of the gates for conducting further research in this field with better-designed studies. Similarly, there is a lack of consensus regarding quality indicators that set the objectives for the program standards for future evaluation. Correspondingly, with the advances in terms of reliance on IT in healthcare, there are promising, but not sufficiently explored, opportunities to expand that aspect for the better delivery of ASPs’ aimed objectives. 

## Data Availability

All utilized data are available online from academic search engines.

## Supplementary Materials

The following supporting information can be downloaded at: https://www.mdpi.com/article/10.3390/antibiotics13030253/s1, Supplement S1: Outlines of the review protocol.
