# Supplementary material for: Evaluation of Hospital Antimicrobial Stewardship Programs: Implementation, Process, Impact, and Outcomes, Review of Systematic Reviews
Source: Antibiotics (Basel). 2024 Mar 12;13(3):253. doi: 10.3390/antibiotics13030253 (PMC10967423; doi:10.3390/antibiotics13030253)
Supplement: Supplementary file 1 [file antibiotics-13-00253-s001.zip › antibiotics-2858079-supplementary.pdf]

**Study : Evaluation of Hospital Antimicrobial Stewardship Programs: Implementation, process, impact, and outcomes, review of systematic reviews**

Supplement S1 : Outlines of the review protocol

**Search period** : 28/08/2022 for the preceding 10 years.

**Search engines** : Five major databases were searched: OVID-Medline, PubMed, Embase, Cochrane, and Google Scholar.

| **Stem** | **AND** | **OR** |
|---|---|---|
| Antimicrobial Stewardship Programs<br>AMSP<br>ASP | Secondary care/ Tertiary care<br>Hospital | Healthcare |
| Antimicrobial consumption<br>Antibiotics | Secondary care / Tertiary care<br>Hospital | Tertiary care<br>DDD |
| Major ASP Concepts | Process<br>Efficacy<br>AMR<br>Economic / Cost effectiveness<br>Safety | Outcome<br>Effectiveness/efficiency<br>Resistance<br>Impact/burden<br>Adverse events |
| Boolean MeSH variations | Antibio*Antimicrob*<br>Steward*<br>ASP*<br>AMSP*<br>AMR*<br>Effic* | Hosp*Hos*<br>Secondary care / Tertiary care |

**Inclusion and exclusion criteria**

| Stem | Inclusion | Exclusion |
|---|---|---|
| Types of studies | Systematic reviews using PRISMA methodology | Non-congruent systematic methodology<br>Reviews<br>Scoping reviews |
| Publication types | Papers published in peer reviewed journals or open access publications | Non peer reviewed articles<br>Policies and guidelines<br>Editorials<br>Letters and comments |
| Research results | Any implementation processes.<br>Any impact<br>Any outcome measures | No available outcomes |
| Settings | Hospital based : Secondary care /tertiary care | Primary care<br>Ambulatory care<br>Long-term facilities / Nursing homes |
| Age | No age restrictions | Nil |
| Gender | All included | Nil |
| Countries | Regions or global | Singular countries |
| Pathogens | Groups / families of pathogens | Singular pathogens<br>Reporting COVID pandemic related outcomes |
| Species | Humans | Other species |
| Language | All languages | Nil |
